# Independent risk factors of mortality in patients with sepsis receiving single-dose etomidate as an induction agent during rapid sequence intubation in a large tertiary emergency department in Thailand

**DOI:** 10.1186/s12873-022-00658-w

**Published:** 2022-06-03

**Authors:** Wasin Pansiritanachot, Onlak Ruangsomboon, Chok Limsuwat, Tipa Chakorn

**Affiliations:** Department of Emergency Medicine, Faculty of Medicine Siriraj Hospital, Mahidol University, 2 Wanglang Road, Bangkok, 10700 Thailand

**Keywords:** Sepsis; Etomidate; Intubation; Emergency

## Abstract

**Background:**

There is limited evidence regarding factors associated with mortality in septic patients receiving etomidate. This study aimed to determine independent pre-intubation factors predicting 28-day mortality in septic patients receiving single-dose etomidate as an induction agent during rapid sequence intubation (RSI).

**Methods:**

This single-center retrospective cohort study included intubated septic patients receiving etomidate as an induction agent during RSI in the emergency department of Siriraj hospital, Bangkok, Thailand, between January 1^st^, 2016 and June 30^th^, 2020. Pre-intubation characteristics were compared between survivors and non-survivors. Independent risk factors associated with 28-day mortality were identified using the Cox proportional hazards regression model. Association between etomidate dosage and mortality was also determined.

**Results:**

A total of 344 patients, 238 (69%) survivors and 106 (31%) non-survivors, were included in the analyses. The initial Cox hazards model identified a pre-intubation lactate level ≥ 4 mmol/L as an independent factor associated with mortality (adjusted Hazards ratio [aHR] 2.66, 95% confidence interval [CI] 1.55–4.56). After removing lactate level from the model due to limited lactate values in the data, cancer was also predictive of 28-day mortality (aHR 1.83, 95%CI 1.10–3.04), while patients with respiratory infections and underlying chronic lung disease were associated with lower mortality (aHR 0.54, 95%CI 0.37–0.80 and aHR 0.57, 0.33–0.96, respectively). Etomidate dosage was not associated with mortality in our study.

**Conclusion:**

In septic patients who received a single dose of etomidate, a pre-intubation lactate level ≥ 4 mmol/L and cancer were associated with increased 28-day mortality, while respiratory infection and underlying chronic lung disease were associated with lower mortality. Physicians may take these factors into consideration when selecting induction agents for septic patients.

## Background

Etomidate has been widely used as an induction agent for rapid sequence intubation (RSI) in the emergency department (ED) because of its minimal effect on blood pressure and heart rate, compared with other available induction agents such as midazolam, propofol, and ketamine [1]. This makes etomidate a compelling sedative agent for intubation when encountering hypotensive patients with concurrent tachyarrhythmia. More importantly, such conditions are frequently observed in sepsis and septic shock patients requiring intubation [2].

A major concern in using etomidate is its inhibition of 11β-hydroxylase, which is an enzyme responsible for converting cholesterol into cortisol. Consequently, the patients are exposed to the risk of having adrenal insufficiency for at least 24–48 h after administration [3], which can theoretically translate into increased mortality in septic patients [4].

There were three meta-analyses published in the past ten years demonstrating a strong association between single-dose etomidate and adrenal insufficiency. Two of them displayed a significant correlation between etomidate and mortality in septic patients [5, 6]. However, the latest meta-analysis in 2015 disproved the association between etomidate and an increase in mortality, promoting the safety of etomidate use [7]. The conflicting results have raised awareness and concerns among emergency physicians regarding the utilization of etomidate as an induction agent in sepsis [8].

Currently, there is scarce evidence defining subgroups of septic patients which might be inflicted by the use of etomidate. Therefore, this study was conducted to identify potential pre-intubation risk factors associated with mortality among septic patients receiving etomidate as the induction agent during RSI.

## Methods

### Study design and setting

This study was a single-center retrospective cohort study conducted in the non-trauma ED of Siriraj hospital, a 2,000-bed university tertiary hospital in Bangkok, Thailand. The ED has more than 20,000 visits per year. All of the ED patients were critical patients with emergency severity index level 1 and 2, who require immediate life-saving interventions or have unstable vital signs [9]. The study was conducted between January 1^st^, 2016 and June 30^th^, 2020. In our ED, all patients with respiratory failure would receive intubation by RSI in the absence of contraindications. Etomidate, ketamine, propofol, and midazolam were readily available as choices of induction agents. The use of etomidate depended on the decision of the attending physicians. Data of all intubated patients in our department were recorded in the written intubation forms, provided with patient identification, the choice of induction agents, an indication for intubation, peri-intubation vital signs, and dosage of the induction agent. After disposition, all ED-visiting patient data were registered into the electronic medical record system, including diagnoses categorized in International Classification of Diseases, 10^th^ revision (ICD-10) codes. In our center, when intensive care units (ICUs) were fully occupied, intubated septic patients would be admitted to general wards in a designated area where intensive care could be provided, and later transferred to the ICUs when available and indicated. This research was written in accordance to the Strengthening the Reporting of Observation studies in Epidemiology (STROBE) guideline [10].

### Participants

All adult (aged over 18 years) septic patients who were intubated by RSI using single-dose etomidate in the ED were included in this study. Every intubated patient receiving etomidate was consecutively reviewed. The final diagnosis of sepsis in the discharge summary records made based on the Sepsis-3 criteria was used to define septic patients in our study [11].

Exclusion criteria were as follows: etomidate given for a surgical procedure, pregnancy, advanced-stage cancer, patients with do-not-resuscitate (DNR) orders (including patients who refused vasopressors, inotropes, hydrocortisone, or antibiotics), patients who refused surgical procedures, patients transferred to other hospitals within 48 h after receiving etomidate, and patients with missing both the primary and secondary outcomes of the study.

### Data collection

Data of patients meeting the inclusion criteria were consecutively reviewed and extracted from the electronic medical record following the suggested methodologic standards in medical record review [12]. The data abstractor was a third-year emergency medicine resident (WP), trained by the attending emergency physician researchers (OR, CL, and TC). Pre-intubation variables, other baseline characteristics, and outcomes were collected in the data abstraction forms. The abstractor’s performance was monitored, with 20% of the records randomly reviewed by the attending emergency physician researcher (TC) for completeness and reliability. Missing values were reported to address the size of potential information bias. There was 100% interobserver reliability of the variables of interest for this cohort since all of the variables could be objectively identified in the electronic medical records.

Pre-intubation variables included age, sex, body weight, comorbidities, history of steroids or herbal use within 3 months, history of adrenal insufficiency, concurrent oral steroid use, sources of infection, concurrent surgical conditions, the presence of hypotensive episodes before intubation, a pre-intubation lactate level, corticosteroids prescription for any reasons before intubation, and etomidate dosage.

Other baseline characteristics and postintubation variables, including Acute Physiology and Chronic Health Evaluation II (APACHE II) score, and intensive care unit (ICU) admission were collected as potential confounders. However, they were not included in the multivariable models since our main focus was on factors that we could determine before intubation.

### Outcome measures

The study primarily aimed to identify potential predictors of poor clinical outcomes in septic patients receiving etomidate as the induction agent for RSI. The primary outcome was independent factors associated with mortality within 28 days after intubation. The secondary outcomes were independent factors associated with the occurrence of any hypotensive episodes that occurred within 48 h after intubation, and any requirements of both vasopressors and hydrocortisone within 48 h after intubation. A hypotensive episode was defined as a mean arterial pressure (MAP) < 65 mmHg that required further interventions, as this cut-off blood pressure is the recommended initial target MAP for septic shock resuscitation [13]. The requirement of both vasopressors and hydrocortisone signified that hemodynamic stability could not be achieved by a certain dose of a vasopressor, thus requiring hydrocortisone for potential adrenal insufficiency [13]. Associations between etomidate dosage and 28-day mortality and the secondary outcomes were also evaluated.

### Statistical analyses

The mortality rate of septic patients who received etomidate was approximately 40% [14, 15]. With presumed 14 pre-intubation risk factors and at least 10 outcome events per variable in the regression model by the rule of thumb [16], a total of 350 patients was required.

Data were expressed as mean and standard deviation (SD) or median and interquartile range (IQR) for continuous variables, and count and percentage for categorical variables. Continuous variables were compared using an independent t-test or the Mann–Whitney U test as appropriate. Chi-square or Fisher exact test was employed as appropriate for comparing categorical variables.

Complete case analysis was performed. We did not employ any imputation techniques to handle missing data because most of the missing data were trivial (< 5%). As for lactate levels, the proportion of patients with missing values was very large (52%) and it could not be assumed to be missing at random. Therefore, we considered the complete-case analysis as the most appropriate.

Univariate Cox proportional-hazards regression analyses were performed to identify an association between each pre-intubation factor and the primary outcome. Post-intubation patient characteristics were not included in the regression model. Multicollinearity among pre-intubation variables was checked using variance inflation factors (VIFs). Strong collinearity was defined when a VIF was more than 4. Results are presented as Hazards ratio (HR) along with its 95% confidence interval (CI), and the corresponding *p*-value. A pre-specified *p-*value < 0.15 was set for purposeful selection of variables in logistic regression [17]. Any pre-intubation variables with a *p*-value < 0.15 were included in the backward stepwise multivariate Cox proportional-hazards regression models to evaluate independent risk factors associated with the primary outcome. The associated factors obtained using these multivariate models were presented with their adjusted HRs (aHR), 95%CIs, and *p*-values. Should any factors contain considerable missing values, multivariate regression models excluding those factors would be re-calculated and reported in different sets of aHR, 95%CIs, and *p*-value. Likewise, for the secondary outcomes, univariate and multivariate logistic regression analyses were performed in a similar manner with the results reported as odds ratios (OR) and adjusted OR (aOR).

For all statistical analyses, a *p*-value of less than 0.05 was considered significant unless defined otherwise. All analyses other than that of the primary outcome should be considered as exploratory analyses, as they might not have enough power since no proper sample size calculation was performed. PASW Statistics for Windows, Version 18.0. (SPSS Inc., Chicago, IL) was obtained for all statistical analyses.

## Results

### Characteristics of patients

During the study period, a total of 415 septic patients received etomidate as an induction agent for RSI; of which 71 were excluded (11 for DNR orders, 15 for multiple-dose etomidate, 17 for advanced-stage cancer, 29 for transferal to other hospitals within 48 h, 1 for refusing surgery, and 2 for missing both the primary and secondary outcomes of the study; of these, 4 were excluded for more than one reason). Ultimately, a total of 344 patients were included in the analysis. Of these, 106 (30.8%) patients had 28-day mortality and were categorized as the non-survivor group with a median survival duration of 6 days (interquartile range 1, 14), while the remaining 238 (69.2%) patients had survived and were classified as the survivor group.

The mean age, sex, body weight, and etomidate dosage between the two groups were similar, as shown in Table 1. However, the proportions of patients with cirrhosis and cancer, and a pre-intubation lactate level were significantly higher among non-survivors, while the proportions of those with underlying chronic lung disease and those with respiratory tract infection as the source of sepsis were higher in survivors. Moreover, the non-survivor group had a significantly higher degree of severity in terms of APACHE II score, as shown in Table 2. Strong collinearity was not observed among pre-intubation variables (maximum VIF = 1.49).

**Table 1:** Pre-intubation characteristics and univariate Cox proportional-hazards regression for associations between pre-intubation characteristics and 28-day mortality

Characteristics	Survivor (*n* = 238)	Non-survivor (*n* = 106)	*p*-value	HR (95%CI)
Age (year), mean ± SD	70.3 ± 15.6	67.1 ± 15.1	0.061	0.99 (0.98,1.00)
Female, n (%)	108 (45.4)	49 (46.2)	0.975	1.01 (0.69,1.47)
Body Weight (kg), mean ± SD	58.7 ± 17.8 (Missing *n* = 8)	59.1 ± 12.1 (Missing *n* = 16)	0.865	1.00 (0.99,1.02)
Comorbidity, n (%)				
Diabetes mellitus	91 (38.2)	42 (39.6)	0.887	1.03 (0.70,1.52)
Coronary artery disease	53 (22.3)	91 (14.2)	0.109	0.64 (0.37,1.11)
Chronic kidney disease	99 (41.6)	41 (38.7)	0.613	0.90 (0.61,1.34)
Immunocompromised state	41 (17.2)	26 (24.5)	0.081	1.48 (0.95,2.31)
Chronic lung disease	75 (31.5)	17 (16.0)	0.004	0.47 (0.28,0.79)
Cirrhosis	24 (10.1)	23 (21.7)	0.008	1.88 (1.18,2.98)
Cancer	17 (7.1)	18 (17.0)	0.010	1.95 (1.17,3.24)
Steroid or herbal use within 3 months, n (%)	59 (24.8)	23 (21.7)	0.601	0.88 (0.56,1.40)
History of adrenal insufficiency, n (%)	3 (1.3)	2 (1.9)	0.498	1.62 (0.40,6.58)
Concurrent oral steroid use, n (%)	26 (10.9)	12 (11.3)	0.731	1.11 (0.61,2.03)
Major sources of infection, n (%)				
Central nervous system	4 (1.7)	3 (2.8)	0.412	1.62 (0.51,5.10)
Respiratory system	168 (70.6)	52 (49.1)	< 0.001	0.48 (0.33,0.71)
Intra-abdominal source	17 (7.1)	13 (12.3)	0.116	1.59 (0.89,2.85)
Genito-urinary system	29 (12.2)	18 (17.0)	0.343	1.28 (0.77,2.12)
Unknown	5 (2.1)	8 (7.5)	0.005	2.79 (1.36,5.74)
Surgical conditions, n (%)	17 (7.1)	11 (10.4)	0.248	1.45 (0.77,2.70)
Presence of hypotension before intubation, n (%)	47 (19.7)	31 (29.2)	0.061	1.49 (0.98,2.27)
Pre-intubation lactate level (mmol/L), mean ± SD	4.9 ± 3.9 (*n* = 95)	9.8 ± 7.7 (*n* = 68)	< 0.001	1.10 (1.07,1.14)
Lactate ≥ 4 mmol/L, n (%)	44 (46.3)	51 (73.9)	< 0.001	2.66 (1.55,4.56)
Corticosteroids prescription for any reasons before intubation, n (%)	42 (17.6)	11 (10.4)	0.111	0.60 (0.32,1.12)
Etomidate (mg), mean ± SD	12.3 ± 2.3 (Missing *n* = 1)	12.4 ± 2.3 (Missing *n* = 1)	0.622	1.02 (0.94,1.11)
Etomidate (mg/kg), mean ± SD	0.22 ± 0.05 (Missing *n* = 9)	0.22 ± 0.05 (Missing *n* = 17)	0.847	1.49 (0.03,85.88)

Abbreviations: *HR* hazards ratio, *CI* confidence interval, *SD* standard deviation

**Table 2:** Comparison of other characteristics and post-intubation variables between survivors and non-survivors at 28 days

Characteristics	Survivor(*n* = 238)	Non-survivor(*n* = 106)	*p*-value
APACHE II, mean ± SD	21.0 ± 7.1	29.2 ± 8.8	< 0.001
ICU admission, n (%)	99 (41.6)	40 (38.1)	0.543
The occurrence of hypotensive episodes within 48 h after intubation, n (%)	143 (60.1)	90 (84.9)	< 0.001
Time from intubation to hypotension (hr), mean ± SD	6.23 ± 9.43	5.77 ± 7.90	0.696
The requirement of vasopressors and hydrocortisone within 48 h after intubation, n (%)	41 (17.2)	45 (42.5)	< 0.001
Norepinephrine dosage at the time of hydrocortisone infusion (mcg/kg/min), mean ± SD	0.11 ± 0.07	0.17 ± 0.12	0.008

Abbreviations: *APACHE II* Acute Physiology and Chronic Health Evaluation II score, *SD* standard deviation, *ICU* intensive care unit

As for the incidence of the secondary outcomes, both the occurrence of hypotensive episodes within 48 h and the rate of requiring both vasopressors and hydrocortisone were more prevalent in the non-survivor group (both *p* < 0.001). Furthermore, norepinephrine dosage at the time of hydrocortisone initiation was significantly higher in the non-survivor group (*p* = 0.008), as also presented in Table 2.

### Factors associated with 28-day mortality

For the primary outcome, the initial multivariate Cox regression model showed that a pre-intubation lactate level ≥ 4 mmol/L was the only independent risk factor for 28-day mortality in septic patients receiving single-dose etomidate as an induction agent during RSI (aHR 2.66; 95%CI 1.55–4.56; *p* < 0.001). However, only 95 (39.9%) survivors and 68 (64.2%) non-survivors had pre-intubation lactate results. Hence, there were a considerable number of missing values in the regression model. After removing the pre-intubation lactate level from the model, cancer (aHR 1.83; 95%CI 1.10–3.04; *p* = 0.020) was identified as an independent risk factor of 28-day mortality. On the contrary, respiratory tract infection (aHR 0.54; 95%CI 0.37–0.80 *p* = 0.002) and chronic lung disease (aHR 0.57; 95%CI 0.33–0.96; *p* = 0.036) were both independent factors associated with lower 28-day mortality.

### Secondary outcomes

For the secondary outcomes, independent factors associated with the occurrence of any hypotensive episodes within 48 h after intubation were a pre-intubation lactate level ≥ 4 mmol/L (aOR 2.83; 95%CI 1.25–6.42), immunocompromised status (aOR 6.00; 95%CI 1.32–26.96), and the presence of hypotensive episodes before intubation (aOR 4.40; 95%CI 1.54–12.55). The requirement of both vasopressors and hydrocortisone within 48 h after intubation was associated with a pre-intubation lactate level ≥ 4 mmol/L and immunocompromised status (aOR 2.78; 95%CI 1.37–5.67 and aOR 3.13; 95%CI 1.46–6.72, respectively).

After excluding the lactate level from the logistic regression models, the prescription of corticosteroids for any reasons before intubation was the only independent protective factor against developing any hypotensive episodes within 48 h after intubation (aOR 0.17; 95%CI 0.08–0.36). The requirement of both vasopressors and hydrocortisone within 48 h after intubation was also positively associated with intra-abdominal infection (aOR 2.82; 95%CI 1.26–6.30), unknown source of infection (aOR 4.11; 95%CI 1.27–13.33), and the presence of hypotensive episodes before intubation (aOR 1.97; 95%CI 1.12–3.50), while underlying chronic lung disease was found to be a protective factor (aOR 0.51; 95%CI 0.26–1.00).

## Discussion

This study, to the best of our knowledge, is the first that specifically aimed to identify pre-intubation risk factors of mortality in septic patients who were intubated with RSI using single-dose etomidate in the ED. A previous retrospective study conducted in an ED and intensive care units reported that multiple vasopressors and intra-abdominal infection were associated with increased mortality in patients receiving etomidate [18]. However, the study did not clearly distinguish pre-intubation factors from other variables, which may have limited utility in the emergency setting.

Our study could demonstrate that a pre-intubation lactate level ≥ 4 mmol/L and cancer were independent risk factors associated with 28-day mortality; meanwhile, respiratory infection and underlying chronic lung disease were associated with lower mortality when etomidate was used for RSI. However, the independent factors identified in this study could neither imply the superiority of other induction agents over etomidate nor encourage the utilization of etomidate over the others in such conditions.

Concordant with other previous studies [19–22], our results confirmed that pre-intubation lactate level was associated with mortality of septic patients regardless of intubation status and induction agents used for RSI. Conversely, although chronic lung disease and respiratory infection have been identified as predictors of poor outcomes in previous studies [23, 24], our study proved otherwise. Both factors could have been associated with lower mortality because patients with pathologies involving the respiratory system were possibly intubated earlier when their degree of sepsis severity was not as high compared to patients with infection at other sites. Nonetheless, this conflicting result mandates future studies. In particular, studies aiming to identify the most appropriate induction agent for these populations should be prioritized.

Adrenal insufficiency following etomidate administration has been linked to increased mortality rates in both septic shock and critically ill patients [4, 25]. It is still controversial whether corticosteroids should be routinely prescribed after etomidate in general critically ill and septic patients since corticosteroids have not demonstrated any changes in mortality outcomes [26–28]. In our study, we also did not find any benefits of prescribing corticosteroids, most of which were dexamethasone for acute exacerbation of chronic lung disease, before intubation with etomidate in terms of decreased mortality. However, the association between corticosteroids and a lower incidence of hypotension within 48 h was observed in our study. Similarly, Köksal and colleagues reported a significantly higher blood pressure at 24 h in intubated ED patients receiving methylprednisolone and etomidate compared to those receiving etomidate alone [29]. Nonetheless, this possible hemodynamic benefit of corticosteroids requires further studies to determine whether dexamethasone, or other commonly prescribed corticosteroids such as hydrocortisone, can prevent hypotension particularly in septic patients receiving etomidate.

Another proposed adjustment to minimize the adverse effects of etomidate is a reduction of its dosage in hemodynamically unstable patients, which is commonly described in anesthesiology literature as this measure could prevent post-intubation hypotension [30]. However, the evidence is limited. Post-intubation hypotension does not solely depend on the dosage of the induction agents, but the clinical conditions of the patients [31, 32]. With the average etomidate dosage of 0.22 ± 0.05 mg/kg in both groups, we have found no association between etomidate dosage and all the adverse outcomes. In fact, the use of the minimum recommended dose in our setting could have contributed to the lack of association between etomidate dosage and the study outcomes. Nevertheless, we suggest that adequate resuscitation should be more of a concern than merely a dose reduction of etomidate when performing RSI.

## Limitations

This study has several limitations. It was a single-center retrospective cohort study that may have suffered from biases generally present in a retrospective study. Pre-intubation conditions of the patients could have influenced the selection of etomidate, which inevitably resulted in a selection bias of certain characteristics in the population. Furthermore, since it was not a routine to obtain lactate level before intubation, there was a large proportion of patients with missing pre-intubation lactate levels, thus affecting the precision and possibly the magnitude of point estimates from the regression analyses. Also, after eliminating the lactate level from the model, it should be noted that the resultant measures of association between other variables and the outcome could have been overestimated. Further studies, preferably prospectively conducted, with enough outcome events and complete data on important and potential associated factors should be conducted. Lastly, it is even more important to note that it is still unclear whether these independent factors are specific to patients receiving etomidate since many of these factors have also been identified as predictors of adverse outcomes in general septic patients receiving any induction agents [21, 23, 24, 33]. Ideally, further studies comparing etomidate to other induction agents in septic patients should be performed. Until these knowledge gaps are filled, physicians should be aware of these factors when using etomidate for induction.

## Conclusion

This study aimed to identify independent pre-intubation factors associated with mortality in septic patients intubated using etomidate. A pre-intubation lactate level ≥ 4 mmol/L and cancer were independently associated with increased 28-day mortality, while respiratory infection and underlying chronic lung disease were factors associated with lower mortality. Physicians may take these factors into consideration when selecting induction agents for septic patients.

## Data Availability

The datasets generated and/or analyzed during the current study are not publicly available due to the sensitivity of the material, but are available from the corresponding author on reasonable request.
